# Dominance, Biomass and Extinction Resistance Determine the Consequences of Biodiversity Loss for Multiple Coastal Ecosystem Processes

**DOI:** 10.1371/journal.pone.0028362

**Published:** 2011-12-07

**Authors:** Thomas W. Davies, Stuart R. Jenkins, Rachel Kingham, Joseph Kenworthy, Stephen J. Hawkins, Jan G. Hiddink

**Affiliations:** 1 School of Ocean Sciences, Bangor University, Menai Bridge, Anglesey, United Kingdom; 2 The Marine Biological Association of the United Kingdom, Plymouth, Devon, United Kingdom; Dalhousie University, Canada

## Abstract

Key ecosystem processes such as carbon and nutrient cycling could be deteriorating as a result of biodiversity loss. However, currently we lack the ability to predict the consequences of realistic species loss on ecosystem processes. The aim of this study was to test whether species contributions to community biomass can be used as surrogate measures of their contribution to ecosystem processes. These were gross community productivity in a salt marsh plant assemblage and an intertidal macroalgae assemblage; community clearance of microalgae in sessile suspension feeding invertebrate assemblage; and nutrient uptake in an intertidal macroalgae assemblage. We conducted a series of biodiversity manipulations that represented realistic species extinction sequences in each of the three contrasting assemblages. Species were removed in a subtractive fashion so that biomass was allowed to vary with each species removal, and key ecosystem processes were measured at each stage of community disassembly. The functional contribution of species was directly proportional to their contribution to community biomass in a 1∶1 ratio, a relationship that was consistent across three contrasting marine ecosystems and three ecosystem processes. This suggests that the biomass contributed by a species to an assemblage can be used to approximately predict the proportional decline in an ecosystem process when that species is lost. Such predictions represent “worst case scenarios” because, over time, extinction resilient species can offset the loss of biomass associated with the extinction of competitors. We also modelled a “best case scenario” that accounts for compensatory responses by the extant species with the highest *per capita* contribution to ecosystem processes. These worst and best case scenarios could be used to predict the minimum and maximum species required to sustain threshold values of ecosystem processes in the future.

## Introduction

It is expected that biodiversity will continue to decline in the 21^st^ century in terrestrial, marine and freshwater ecosystems [1]. Two decades of research into the role of biodiversity in ecosystem functioning has demonstrated that species and functional diversity can have a positive effect on a variety of key ecosystem processes such as carbon and nutrient cycling [2], [3], [4]. Understanding how such processes deteriorate as species are lost from ecosystems is necessary to enable predictions of the decline in ecosystem processes during extinction events. Translating the results of many biodiversity–ecosystem functioning (BEF) investigations into predictions in the real world is however difficult. This is because a major focus in BEF research has been the use of replacement series designs, in which artificial biodiversity gradients are created from randomly selected species [5], [6], [7], [8]. In addition, initial total organism abundance is often fixed across treatments which makes the *a priori* assumption that extinction resilient species compensate to some extent for the loss of their competitors. The resulting assemblages consequently do not reflect reality where species loss in unlikely to be random [9], [10] and extinction resistant species do not always compensate for biodiversity loss [11], [12].

Ecosystems undergoing non-random extinction can display more rapid or slower declines in ecosystem functioning compared to random species loss scenarios [13], [14], [15], [16], [17], [18], dependent on the extinction resistance of those species which contribute most to ecosystem processes [13], [16]. For example, manipulations of intertidal macroalgae assemblages which simulated extinctions driven by accelerated wave exposure, had a greater impact on nitrogen uptake than random extinctions because species which utilized nutrients at higher rates were also least resistant to wave exposure [13]. While a number of field and laboratory experiments have highlighted how the extinction resistance of species with higher rates of resource use turnover can define BEF relationships, the population abundance of component species is often fixed. In nature, however, species occur in widely contrasting abundances, with a few species often dominating the biomass of an assemblage [19]. In order to understand how ecosystem functioning will respond to species loss in natural communities there is a need to quantify biodiversity ecosystem function relationships under realistic extinction scenarios and in natural ecosystems, where biomass is not equal among species.

The ‘mass – ratio’ hypothesis [20] suggests that dominant species control the majority of ecosystem processes in natural assemblages because species contributions to ecosystem processes correlate closely to their abundance. If this theory is valid, then estimates of species population abundance can be used to predict the short term decline in ecosystem processes with species loss under realistic extinction scenarios. Such predictions represent ‘worst case scenarios’ of biodiversity-ecosystem process relationships because they do not account for compensation by extinction resilient species. This theory is a kin to ‘The Metabolic Theory of Ecology’ which predicts that the turnover of energy by a population will be proportional to the biomass of that population, because species metabolic rates scale with individual biomass in the same way as population density scales negatively with individual biomass [21], [22].

While species contributions to community biomass might predict the functional consequences of species loss in the short term, achieving longer term predictions requires that models account for compensation by extinction resistant species [23]. Although a limited number of studies have modelled density compensation during extinction [24], little empirical evidence is currently available to enable compensation dynamics to be included in real world predictions of biodiversity loss [11]. The absence of being able to predict the long term consequences of biodiversity loss highlights a need for a broad predictive framework which draws on worst and best case scenarios to describe the boundaries of possible BEF relationships in nature.

This investigation explored whether population biomass can be used as a proxy of species contributions to ecosystem processes. Specifically we hypothesised that a mass-ratio relationship existed across assemblages consisting of species within a single trophic level, that would allow biomass to be used to approximate species contributions to ecosystem processes. Estimates of population abundance can then be combined with predictions of an extinction order to provide ‘worst case scenario’ predictions of a biodiversity-ecosystem function relationship. We then describe a simple technique which can be used to quantify a best case scenario of how extinction impacts on ecosystem functioning which accounts for functional compensation by extinction resistant species. Collectively the best and worst case scenarios represent the boundaries of the envelope of possible biodiversity – ecosystem process relationships in an ecosystem undergoing long term species loss. Hence they can be used to estimate the minimum and maximum number of species required to maintain various levels of ecosystem functioning.

### Experimental overview

The species richness of three contrasting marine communities (salt marsh plants, sub-tidal sessile invertebrates, and a macroalgal turf) was manipulated to simulate an extinction scenario that was realistic for each respective assemblage (Table 1, Figure S1). Assemblages were manipulated by removing species in a subtractive fashion so that biomass was allowed to vary naturally with each species lost. Changes in multiple ecosystem processes were then measured across the resulting gradients of species richness. A range of processes important for the functioning of marine coastal ecosystems were measured including gross community productivity (salt marsh plants and macroalgal turfs), uptake of the key nutrients ammonium and nitrate (macroalgal turfs) and clearance rates of micro-algae (sub tidal suspension feeding sessile invertebrates). The resulting species richness–ecosystem function relationships were then used to estimate the contributions of the species in each of the ecosystems to each of the ecosystem processes measured. The ‘functional contributions’ were then related to the amount of biomass each species contributed to the community to test the presence of a mass-ratio relationship which would allow population biomass to be used to approximate the contributions of species to ecosystem processes in nature.

**Table 1:** The rank order of species extinction in response to different disturbances in natural undisturbed marine coastal ecosystems.

Ecosystem	Species	Slope ±95% CI			*F*	*p*	r^2^	Rank ES
Salt marsh plants	*Salicornia ramosissima*	−0.189	±	0.016	_(1,2)_ 25.75	0.037	0.93	1
	*Puccinellia maritima*	−0.071	±	0.030	_(1,4)_ 43.83	0.003	0.92	2
	*Armeria maritima*	−0.060	±	0.020	_(1,4)_ 62.58	0.001	0.94	4
	*Limonium humile*	−0.056	±	0.014	_(1,4)_ 32.04	0.005	0.89	5
	*Plantago maritima*	−0.040	±	0.022	_(1,4)_ 33.82	0.004	0.89	6
	*Aster tripolium*	−0.016	±	0.038	_(1,4)_ 1.155	0.343	0.22	7
	*Triglochin maritima*	0.027	±	0.076	_(1,4)_ 2.223	0.210	0.36	8
Sessile invertebrates	*Scypha compressa*	−0.294	±	0.129	_(1,3)_ 207.6	0.002	0.96	1
	*Sycon ciliatum*	−0.243	±	0.056	_(1,3)_ 147.6	0.000	0.97	2
	*Ascidiella aspersa*	−0.222	±	0.068	_(1,3)_ 196	0.001	0.98	3
	*Mytilus edulis*	−0.085	±	0.062	_(1,4)_ 12.87	0.013	0.69	4
	*Balanus crenatus*	−0.055	±	0.026	_(1,4)_ 113.3	0.002	0.85	5
Macroalgae turfs	*Ectocarpus sp.*	−0.011	±	0.007	_(1,4)_ 18.91	0.012	0.78	1
	*Ulva sp.*	−0.010	±	0.005	_(1,4)_ 34.91	0.004	0.87	2
	*Membranoptera alata*	−0.009	±	0.007	_(1,4)_ 10.97	0.030	0.67	3
	*Fucus serratus*	−0.008	±	0.007	_(1,4)_ 11.52	0.024	0.68	4
	*Gracillaria verrucosa*	−0.005	±	0.006	_(1,4)_ 4.938	0.090	0.44	5
	*Ceramium rubrum*	−0.004	±	0.008	_(1,4)_ 1.391	0.304	0.07	6
	*Chondrus crispus*	0.001	±	0.002	_(1,4)_ 1.516	0.286	0.09	7
	*Cladaphora sp.*	0.039	±	0.037	_(1,4)_ 8.189	0.046	0.59	8

A species extinction order was obtained in the case of each ecosystem by ranking the value of the slope of the 0 standardised relationship (for which values of *F*, *p* and r^2^ are presented) between abundance relative to controls and disturbance intensity in the case of salt marsh plants and duration in the remaining communities (see Figure S1). Salt marsh plants were treated with elevated quantities of fucoid algal mats, sessile invertebrate communities were exposed to hypoxia, and elevated wave exposure was simulated on macroalgal turfs. Rank ES is Rank Extinction Susceptibility where 1 is the most extinction susceptible species. Numbers in parenthesis represent the degrees of freedom of each regression estimate.

In contrast to previous realistic extinction scenario investigations which use either species traits [15], [16], [17] or observational approaches [13], [14], [16] to determine realistic species extinction sequences, we conducted an *in situ* disturbance experiment in each of the previously undisturbed ecosystems to empirically quantify the order in which species disappear when disturbed. In the salt marsh we simulated the effect of an increase in the quantity of algal mat deposited on the plant assemblage while in the macroalgal turfs we simulated an increase in wave exposure. The frequency and intensity of both of these disturbances are likely to impact these communities more with predicted increases in the frequency and severity of storms in the North East Atlantic [25] and have been shown to significantly alter the structure of those communities where they are prevalent [26], [27]. To obtain a realistic extinction order for the sessile suspension feeding invertebrates, PVC tiles previously left to colonise naturally over one year were exposed to an acute hypoxic event which simulated the kind of oxygen depletion which has become an environmental issue of increasing concern in coastal and deep sea environments [28], [29]. The sensitivity of each species to disturbance referred to here as ‘extinction resistance’ (quantified as the slope of the relationship between relative species abundance and disturbance duration or intensity; see Figure S1) was then ranked to provide a predicted order of species extinction in response to each of the disturbance events mimicked. Using this approach, extinction resistance was estimated for those species which were sufficiently abundant and homogenous in their distribution (Table 1).

**Figure 1:**
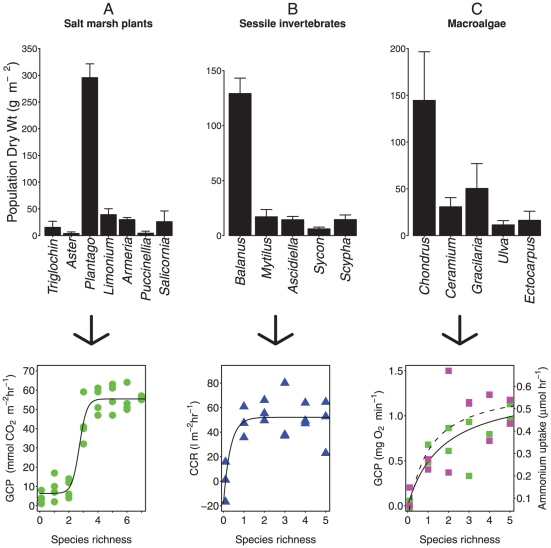
The short term impact of species loss on ecosystem processes can be described from the order of species extinction with respect to their population biomass. Bar charts represent the average population dry weight (error bars represent 95% confidence intervals) of constituent species in natural communities, presented in order of their extinction position (right to left) correspondent to each of the species richness-ecosystem process relationships below. Gross Community Productivity (GCP) in salt marsh plants (•, green) declined in a sigmoidal fashion with species loss because the dominant species was not the most or least resistant to extinction. Community Clearance Rates (CCR) of microalgae in the sessile invertebrate assemblage (▴, blue), and Gross Community Productivity (GCP) (▪, green) and Ammonium uptake (▪, magenta) in intertidal macroalgae did not respond rapidly to species loss because the dominant species was the most resistant to extinction. The influence of dominance structure and extinction order on the resulting biodiversity ecosystem function relationships is explained by the 1∶1 ratio between biomass contribution and functional contribution described by figure 2.

Natural communities (community composition presented in Figure 1) were manipulated by removing species sequentially in a subtractive fashion to simulate the previously derived extinction orders. Changes in ecosystem processes were then quantified across the resulting gradient of species richness. Because biomass was allowed to vary with each species removed, and ecosystem processes were measured instantaneously (i.e. in the absence of density compensation), the derived relationships represented worse case scenarios of the rate of depletion in ecosystem processes with species loss. A range of linear and non-linear models (linear, exponential, asymptotic, Michelis-Menten, and log logistic) were fitted to the resulting species richness-ecosystem process relationships and the most parsimonious model describing each relationship selected using Akaike's Information Criterion (AIC). AIC is parsimonious in that it maximises goodness of fit while minimising the number of model parameters. The significance of the model fits was checked by comparison to a null (intercept only) model. Species richness was considered to not have an impact on an ecosystem process where no model was found to be significantly different from the null model.

## Results

### The response of ecosystem processes to species removal

All but one of the ecosystem processes measured (nitrate uptake in macroalgal turfs) were significantly affected by species loss (Figure 1, Table 2). Gross community productivity declined in a sigmoidal (log-logistic) fashion with species loss in the salt marsh plant assemblage (Figure 1A, Table 2), indicating that species which contributed most to primary production were intermediate in their extinction resistance. The decline in gross community productivity from ∼50 to 10 mmol CO_2_ m^−2^ hr^−1^ between species richness 3 and 2 coincided with the loss of the most dominant species, *Plantago maritima*, which contributed to 71% of the community biomass (measured as dry above ground biomass) of the salt marsh plant assemblage (Figure 1A). In the sessile invertebrate assemblage community clearance rates of micro algae declined in an asymptotic curve with species loss (Figure 1B, Table 2), indicating that species which contributed most to clearance rates were most resistant to extinction. This relationship appeared heavily influenced by the 0 species treatment (a bare PVC tile only) because the dominant species (*Balanus crenatus*) which contributed to 71% of community biomass (measured as dry tissue weight) was the most resistant to hypoxic disturbance, and the removal of more extinction susceptible species therefore had little detectable impact on community clearance rates. Both gross community productivity and ammonium uptake declined in a Michelis-Menten curve as species were lost in the macro algal turf assemblage (Figure 1C, Table 2), indicating that species which contributed most to both of these processes were more extinction resistant. These species included the dominant and most resistant species *Chondrus crispus* (57% of community biomass measured as total dry weight), and the two sub-dominant species *Gracilaria verrucosa* and *Ceramium rubrum* (20% and 12% of community dry biomass respectively). In all of the ecosystems studied the biomass dominant species appeared to be conducting the majority of the ecosystem processes being performed, and the biodiversity-ecosystem process relationship was dependent on the extinction resistance of these species (Figure 1). In addition, assemblages in which biomass is distributed more evenly, such as the macroalgal turfs, displayed less saturating and more linear species richness–ecosystem functioning relationships (Figure 1C) further indicating that biomass was central in determining the contribution of species to ecosystem processes.

**Table 2:** Output from the model selection procedure applied to species richness-ecosystem process relationships.

Model	Salt marsh plants	Sessile invertebrates	Macroalgae turfs		
	GCP	CCR	GCP	NH_4_^+^ uptake	NO_3_^−^ uptake
**Linear**	250.7***	166.0*	4.7**	−8.4*	−12.7
**Exponential**	264.7***	167.1	6.9**	−6.9*	−11.9
**Michaelis−Menten**	244.8***	159.3**	1.6***	−11.7**	NO FIT
**Asymptotic**	245.4***	153.8***	4.0**	−10.4*	NO FIT
**Logistic**	213.3***	NO FIT	NO FIT	−9.4*	NO FIT

Values are of Akikes' Information Criterion (AIC) where smaller values indicate a more parsimonious fit of the model to the data.. Models which were significantly different from a null (intercept only) model are denoted by stars (*** = *p*<0.001, ** = *p*<0.01, * = *p*<0.05). Where no model was found to be significantly different from the null, it was concluded that species loss had no impact on that ecosystem process (NO_3_^−^ flux only). Of the models which were significantly different from the null, that with the lowest AIC value was selected as the model which best described the relationship between species richness and an ecosystem process (underlined). GCP = Gross Community Productivity, CCR = Community Clearance Rate. NO FIT = The model insufficiently described the pattern of the data for it to be fitted statistically.

### The mass-ratio relationship

**Figure 2:**
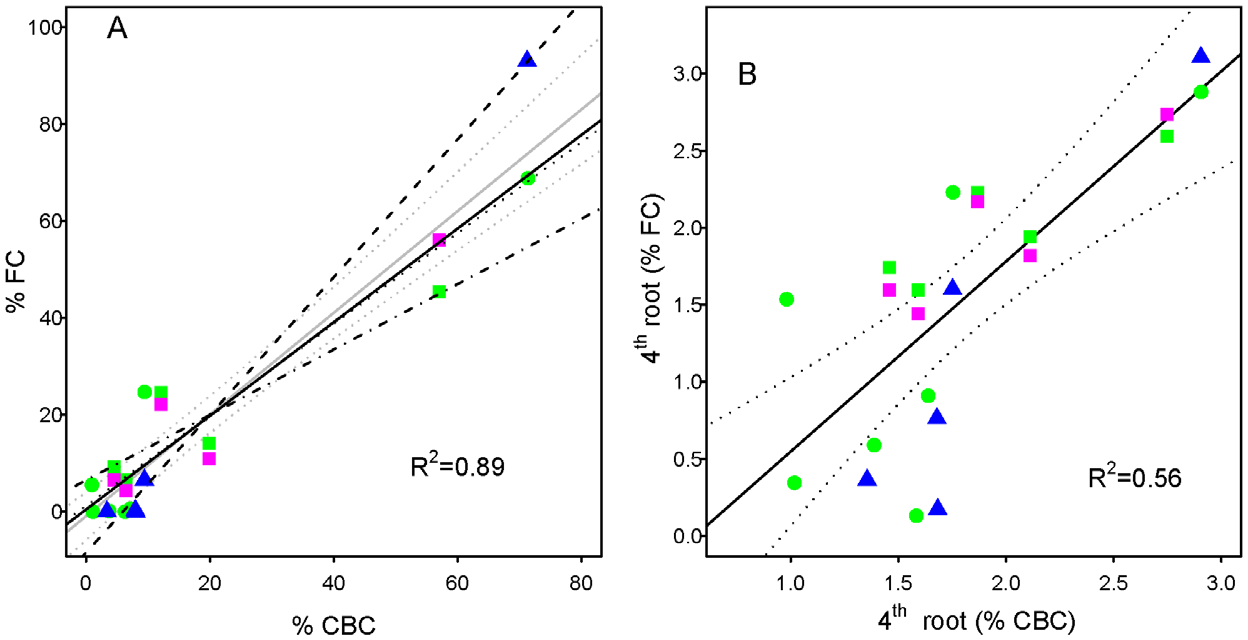
The Mass-Ratio relationship in multiple marine communities. **A**. Species % Contributions to Community Biomass (% CBC) describe 89% of the variability in their % contributions to different ecosystem processes (% Functional Contribution, FC) across contrasting ecosystems (grey solid line with dashed grey 95% confidence intervals), however this relationship was significantly different for different ecosystems. Solid line = gross community productivity in salt marsh plants, dashed line = community clearance rate in sub tidal sessile invertebrates, dotted line = gross community productivity in macroalgae turfs, dashed dotted line = ammonium uptake in macroalgae turfs). **B**. Following 4^th^ root transformation to remove the leverage of dominant species, the relationship was independent of the ecosystem or ecosystem process being studied. Solid lines represent significant regression fits with dotted lines representing their 95% confidence intervals. Symbol and colour assignment is the same as Figure 1.

These results indicated that biomass may be useful for approximating the contribution of species to key processes in natural ecosystems. To test this hypothesis, where species richness was found to have a significant influence on an ecosystem process, the contribution of each species to that process (functional contribution, FC) was estimated as the difference in the fitted values of the selected models (presented in Figure 1) between the two treatments where that species was lost. These values were then standardised to percentages of the summed value of all contributions to an ecosystem process (i.e. the maximum rate at which a process is being performed) in each community to provide values of the percentage that species contributed to an ecosystem process (Functional Contribution, % FC). To test whether population biomass can approximate the contribution of species to an ecosystem process, the % contributions of all species in all ecosystems were then related to their respective % contributions to community biomass (Community Biomass Contribution, % CBC) (Figure 2).

The % contribution of the salt marsh plants to gross community productivity was 0.97 times their % contribution to community biomass (*F_1,5_* = 51.05, *p*<0.001); the % contribution of the sub tidal sessile invertebrates to community clearance rate was 1.42 times their % contribution to community biomass (*F_1,3_* = 640.2, *p*<0.0001); the % contribution of macro algae to gross community productivity was 0.68 times their % contribution to community biomass (*F_1,3_* = 16.79, *p*<0.05); and the % contribution of macroalgae to ammonium uptake was 0.94 times their % contribution to community biomass (*F_1,3_* = 26.87, *p*<0.05) (Figure 2A). When this relationship was tested for all the data pooled together 89% of the variance in % FC was explained by % CBC in a 1∶1 ratio (% FC∼1.05*% CBC, *F_1,20_* = 163.5, *p*<0.0001) (Figure 2A). While this relationship was not significantly different between different ecosystem processes (ANCOVA, *F_1,14_* = 1.26, *p* = 0.281), it was significantly different between ecosystems (ANCOVA, *F_2,14_* = 6.85, *p*<0.01). Dominant species, however, had a strong leverage effect on this analysis (seen by the clustering of rarer species in the bottom left of Figure 2A), and experimental variability in the estimated contribution of dominant species to ecosystem processes could have resulted in the observed difference in the % FC∼% CBC relationship between the different ecosystems. Indeed only the relationship between % community clearance rate and % community biomass in the sessile invertebrate assemblage (Figure 2A, black dashed line) lay outside the 95% confidence intervals of the relationship between % FC and % CBC obtained for all data pooled together. This indicated that one species in the sub tidal invertebrate assemblage, the dominant species *Balanus crenatus*, could be responsible for the difference in the relationship between % FC and % CBC between different ecosystems. Hence both % FC and % CBC were 4^th^ root transformed to remove the leverage of dominant species, while maintaining the linearity of the relationship. Following 4^th^ root transformation the 1∶1 relationship between % functional contribution and % community biomass contribution remained significant (% FC = 1.23*% CBC, *F_1,20_* = 27.97, *p*<0.0001) (Figure 2B). The slope of the 4^th^ root transformed relationship was not significantly different between the different ecosystems (ANCOVA *F_1,14_* = 0.41, *p* = 0.532) or ecosystem processes (ANCOVA *F_2,14_* = 1.33, *p* = 0.300) indicating that the 1∶1 relationship between % FC and % CBC consistently underpinned each of the biodiversity-ecosystem process relationships despite differences in both their species composition and dominance structure.

These results suggest that a 1∶1 ‘mass–ratio’ relationship exists between the biomass a species contributes to an assemblage, and the contribution of that species to an ecosystem process. Hence population biomass could be used to approximate the contribution of species to ecosystem processes. The instantaneous or worst case scenario decline in ecosystem processes with species loss could therefore be predicted provided the order of species extinction with respect to their population biomass is predictable.

### Predicted scenarios of species richness–ecosystem process relationships

Over longer timescales, compensation of ecosystem processes through increases in the biomass of more extinction resistant species may compensate for the loss of species which are more sensitive to extinction. However, species do not always compensate for one another in natural ecosystems [11] making it difficult to make accurate predictions of whether compensation by extinction resilient species will ameliorate the impact of species loss on ecosystem processes. Predicting the upper and lower boundaries of possible species richness–ecosystem process relationships, however, provides a way for conservation managers to envisage the range of responses they can expect to observe in an ecosystem process as species are lost from an assemblage. In order to predict the upper and lower boundaries of possible species richness–ecosystem process relationships we defined two scenarios, a worst case and a best case scenario. Under both scenarios we modelled the decline in ecosystem processes as species were lost in the extinction sequence previously observed during the disturbance experiment. In the worst case scenario, extinction resilient species do not compensate for the loss of their competitors during extinction. This is equivalent to the removal experiments conducted in the current study, hence we used the observed species loss–ecosystem process relationships as our worst case scenario predictions. In contrast to the worst case scenario predictions which do not account for functional compensation by extinction resistant species, in the best case scenario the extant species with the highest *per capita* (unit biomass) contribution to ecosystem functioning were assumed to fully compensate for the loss of biomass associated with each extinction. The rate of an ecosystem process was then estimated by multiplying the population biomass of the remaining species at each stage of community disassembly, by their respective *per capita* (unit biomass) contributions to that process, and subsequently summing the resulting values. The resulting relationship represented a best case scenario because 1) compensation was modelled using the species with the highest *per capita* contribution to ecosystem processes, 2) it was assumed that species compensated to maintain a constant level of biomass in the ecosystem, and 3) reductions in ecosystem processes resulting from the loss of complementary or facilitative interactions between species were not taken into account. The worst and best case scenarios of density compensation could then be used to define the maximum and minimum number of species required to sustain various fractions of an ecosystem process.

**Figure 3:**
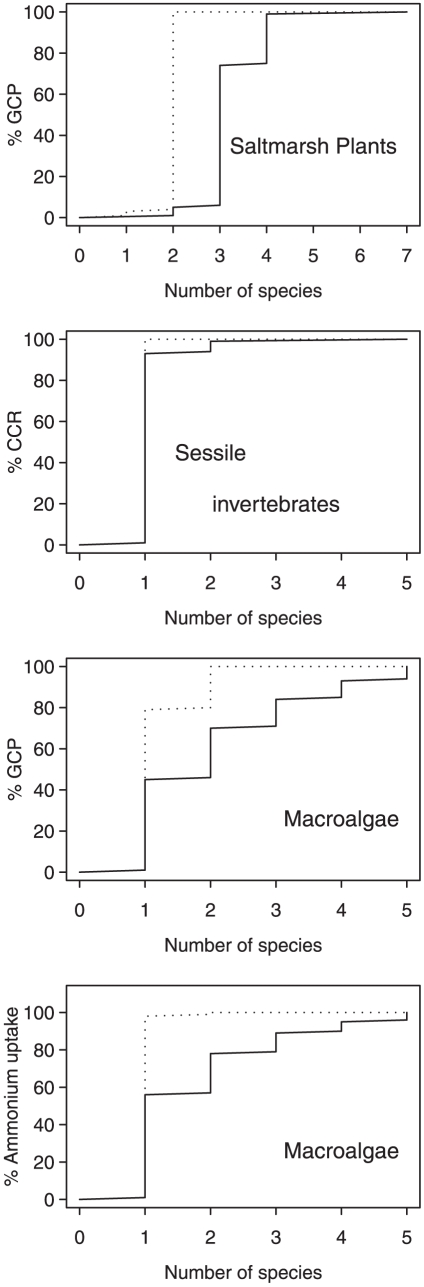
Worst and best case scenarios of the impacts of extinction on ecosystem processes in contrasting marine communities. Worst case scenarios (solid lines) represent the maximum number of species required to maintain different levels of ecosystem processes, while best case scenarios (broken lines) represent the minimum number of species required to maintain different levels of ecosystem processes. Results are predicted for a salt marsh plant assemblage exposed to climate driven elevations in fucoid algae deposition, a sessile invertebrate assemblage exposed to acute hypoxia and an assemblage of intertidal macroalgae exposed to climate driven impacts from wave exposure. GCP = Gross Community Productivity, CCR = Community Clearance Rate.

In Figure 3 the number of species required to sustain ecosystem functioning from 0 to 100% is presented. Under the best case scenario, compensation reduced the number of species required to maintain ecosystem functioning in the majority of ecosystems. The scope for compensation is reduced, however, where species which strongly dominate ecosystem processes are most resistant to extinction. For example in the sessile invertebrate assemblage, one species sustained >90% of community clearance rate in the absence of density compensation, while in the macroalgae assemblage, compensation by the most extinction resistant species sustained >90% of ammonium uptake, whereas four species were required to maintain this level of functioning in the absence of density compensation (Figure 3). The two most extinction resistant salt marsh species can maintain full (100%) gross community productivity where this would require four species in the absence of density compensation (Figure 3).

## Discussion

The results of this investigation suggest that the functional contributions of species can be approximated from their contributions to community biomass in both floral and faunal assemblages. The worst case impact of species loss on ecosystem processes can therefore be predicted when the order of extinction and the population biomass of species is known. We found that ecosystem processes were relatively insensitive to low levels of species loss for the majority of the ecosystems investigated because the biomass of most ecosystems was strongly dominated by one species, and that species was consistently among the most resistant to extinction. However, dominant species are often targeted during exploitative activities such as fisheries [30], and deforestation [31]. These results suggest that where dominant species are being exploited, key ecosystem processes such as carbon fixation in forests and nutrient cycling by fish may be deteriorating at rapid rates. Hence the value of dominant species should not be underestimated [32].

The link between biomass and productivity has been previously demonstrated in terrestrial plants [33], [34]. Its wider application to alternative ecosystems and ecosystem processes, and the potential usefulness it has for the prediction of biodiversity-ecosystem process relationships has, however, not previously been explored. Computer simulations of nutrient fluxes in multi-trophic fish assemblages [17] and bioturbation in sub-tidal macro-invertebrates [16] suggest that the mass-ratio relationship may be important for driving the short term responses of ecosystem processes to species loss in widely contrasting ecosystems. Whether the mass-ratio correlation can be applied to alternative ecosystem functions such as crop pollination, secondary productivity and habitat provision is however uncertain. While a link between biomass and ecosystem processes which are strongly influenced by organism physiology seems intuitive, the strength of the relationship when considering the added complexity of, for example, trophic interactions is uncertain. For example, while we found no evidence that species with low population biomass contributed disproportionately to ecosystem processes, the presence of such ‘keystone species’ [35], [36] is not uncommon in natural ecosystems [35], [37], [38], and their impact is often exerted through trophic or facilitative interactions [38], [39]. The mass-ratio relationship is therefore unlikely to be a ‘rule’ which can be universally applied to all species in all ecosystems, but may well be usefully applied to understand how ecosystem processes are distributed across species in unitrophic assemblages.

It is also important to highlight that whilst the mass-ratio relationship stipulates that the rate of decline in ecosystem processes will be highly dependent on the extinction susceptibility of dominant species in the short term, the loss of functional diversity associated with the extinction of rarer species may render communities and hence ecosystem processes more unstable in the face of fluctuating environmental stressors at longer time scales (community stability). While results from artificially assembled communities suggest that species diversity provides greater temporal stability in community biomass [40], recent investigations which experimentally remove species from natural ecosystems suggest that community stability is dependent on the resilience of dominant species to environmental fluctuations [41], [42]. Further research into the relative importance of dominant vs rare species in the maintenance of community stability in natural ecosystems is clearly required.

The mass-ratio relationship suggests that when combined with predicted extinction orders, species abundance data can be used to predict the instantaneous impact of biodiversity loss on ecosystem processes. Such predictions however represent only worst case scenarios of BEF relationships in the real world, because compensation by extinction resistant species is not taken into account. Here, we described a simple technique for estimating a best case scenario in which the impact of species loss on ecosystem processes is ameliorated by an increase in the abundance of the species with the highest *per capita* contribution to an ecosystem process. Collectively the best and worst case scenario curves presented in Figure 3 represent the minimum and maximum number of species required to sustain ecosystem functioning during specific extinction events likely to occur in nature. For example between two and seven species are required to sustain 100% gross community productivity in the salt marsh plant assemblage, while between one and five species are required to maintain 100% of community clearance rate in the sessile invertebrate assemblage (Figure 3). In order to maintain 50% ecosystem functioning between two and three species are required for salt marsh plants, one species for the sessile invertebrate assemblage, between one and two species for gross community productivity in macroalgae, and one species for ammonium uptake in macroalgae (Figure 3). It is important to note that these predictions are specific to the structure of each community and the extinction order with respect to population biomass.

Obtaining similar predictions for alternative ecosystems requires that the distribution of biomass in the assemblage is known, the *per capita* functional contribution of each species is known, and the order of species extinction is known. It should also be highlighted that these worst and best case scenarios represent the boundary of possibilities of the true long term biodiversity–ecosystem process relationship. Positive biodiversity effects such as niche complementarity operating at longer time scales, can be expected to increase the number of species required to maintain an ecosystem process above that predicted by the best case scenario but below that predicted by the worst case scenario. This is because the best case scenario assumes that increases in intraspecific competition associated with species loss does not affect the maximum standing biomass of an ecosystem, where in reality such increases in competitive interactions have been shown to decrease the maximum standing biomass of plant assemblages in many large scale biodiversity-ecosystem functioning investigations [5], [43]. While the best case scenario provides a useful tool for defining the minimum number of species required to sustain ecosystem processes, compensation by extinction resistant species may not result in full recovery of community biomass, and could result in an increase in community biomass. Predicting the true number of species required to maintain ecosystem processes in the long term requires a better understanding of how extinction resistant species compensate for the loss of their competitors in nature, and the factors on which such interactions can be dependent.

This study demonstrates evidence of a link between biomass and ecosystem processes which is potentially useful for predicting the short term consequences of species loss for ecosystem processes in unitrophic assemblages. Further research is required to establish the wider relevance of this link to alternative types of ecosystem functioning and more complex ecosystems. While achieving accurate predictions of species richness-ecosystem process relationships which can account for density compensation is currently difficult, the worst and best case scenarios presented here provide one approach to estimating the range of negative impacts that loosing species can have on key processes in an ecosystem. Future research which focuses on understanding compensatory interactions between extinction resistant and susceptible species in the real world could provide useful insights for predicting more realistically the consequences of extinction in natural communities for ecosystem functioning.

## Methods

### Experimental Communities

All experimental communities were located on or around the Isle of Anglesey, UK. The salt marsh plant assemblage was a previously undisturbed marsh located in the Cefni Estuary (53° 10′ 12″ N: 4° 23′ 39″ W). The plant assemblage consisted of the perennials *Plantago maritima*, *Limonium humile*, *Armeria maritima*, *Aster tripolium*, *Triglochin maritima*, *Puccinellia maritima*, *Atriplex portulacoides* and *Spergularia media*, and the annual *Salicornia ramosissima*. However *Atriplex* and *Spergularia* were uncommon and patchily distributed species preventing their abundance from being reliably estimated. These species were therefore omitted from our simulated extinction scenario, as their extinction resistance could not be reliably quantified. The sessile invertebrate assemblage was colonised on roughened grey PVC tiles deployed in the Menai Strait (53° 13′ 46″ N: 4° 09′ 10.44″ W) from April 2008 to January 2009. The resulting assemblage was dominated by the barnacle, *Balanus crenatus*. The sponges *Scypha compressa* and *Sycon ciliatum*, the ascidian *Ascidiella aspersa*, and the bivalve mollusc *Mytilus edulis* were also consistently present on each tile All of these species are sessile suspension feeders which intercept and feed on microalgae and particulate organic matter in the water column. A number of less common species occurred less frequently in the assemblage, however , reliable estimates of the extinction resistance of these species could not be made.The macroalgal turf community was located at the subtidal fringe of an intertidal boulder field located on a sheltered shore near Penmon Point (53° 17′ 59″ N: 4° 03′ 04″ W). The community was dominated by the three red algae *Chondrus crispus*, *Ceramium rubrum* and *Gracilaria verrucosa*. Ephemeral species which were also present, included the brown algae *Ectocarpus* sp., and the green algae *Ulva* sp. The extinction resistance of a number of other species was estimated, however these species could not be included in the extinction scenario manipulation due to limits on replication. These were the less common species, *Fucus serratus*, *Membranoptera alata*, and *Cladophora* sp.

### Quantifying the Extinction Order

A realistic sequence of species extinction was quantified in each ecosystem by mimicking realistic disturbance events. Salt marsh plants were exposed to five different quantities of fucoid algal mat netted onto 1 m^2^ plots for 60 days. Treatments consisted of 0, 3, 6, 9, 13 l m^−2^ wk^−1^, with five replicates in each treatment except 0 which contained ten replicates. Following disturbance % cover of each species was quantified using a 0.25 m^2^ 49 point quadrat placed centrally within each plot.

The sessile invertebrate community was exposed to hypoxia for different time periods. Hypoxia was generated by removing colonised tiles and sealing them inside polyethylene bags filled with seawater. Bags were then placed in free flowing seawater from the Menai Strait under laboratory conditions. Communities were exposed to hypoxic conditions for 0, 1, 3, 4 and 7 days (n = 4 per treatment). O_2_ concentrations in the bags decreased exponentially to 22, 7, 5 and 2% of natural seawater O_2_ concentration in the Menai Strait on the 1st, 3rd, 4th and 7th days of disturbance respectively. Following hypoxic treatments tiles were placed back in the Menai Strait for seven days to allow deceased individuals to decay allowing them to be reliably differentiated from living individuals. The population wet tissue weight of each species was then quantified across all tiles.

Macroalgae communities were disturbed for two minutes using a high pressure hose to mimic an increase in wave impact velocity. This disturbance was repeated on the lowest spring tide of each month from 27/02/2010 to 26/04/2010 (4 disturbances total) to simulate an increase in the frequency of wave impact events at this intensity. Five 0.09 m^2^ plots were disturbed while five control plots were left undisturbed. % cover of each species was recorded in all plots prior to each disturbance event using a 16 point intercept quadrat. Hence % cover was recorded following 0, 1, 2, 3 and 4 disturbances.

Abundance measures were converted to relative abundance for all ecosystems by dividing by the average of the control treatment (where no disturbance was simulated) and subtracting 1. In forcing the relationship between average relative abundance per treatment and disturbance through 0, the slope of the regression estimate becomes a comparable measure of extinction resistance between species (Table 1, Figure S1). Regressions were performed between relative % cover and quantity of algae deposited in the salt marsh, relative wet tissue weight and time in the sessile invertebrate communities, and relative % cover and time in the macroalgae communities. Where the abundance of a species declined to 0 in low disturbance treatments (*Salicornia* for salt marsh plants, *Scypha compressa* and *Sycon ciliatum* for the sessile invertebrate assemblage), responses from higher disturbance treatments were excluded from the analysis to prevent further 0 abundance measurements underestimating the regression slope estimate and hence extinction susceptibility (see Figure S1). Residual normality was checked using the Shapiro-Francia test. One data point for *Ascidiella aspersa*, day 2 of hypoxia, was omitted from the analysis following verification of incorrect identification of deceased individuals by reference to previously archived photographs of tiles taken following disturbance.

### Simulating extinction

Natural communities were manipulated to simulate the order of extinction observed from the disturbance experiments (Table 1, Figure S1). Species were removed in a subtractive fashion so that each experimental unit represented a progressive stage of community disassembly in the absence of density compensation (n = 4 per treatment for salt marsh plants, n = 3 for the sessile invertebrate assemblage, n = 2 per treatment for macroalgae turfs). Because practically we could only quantify the functional contribution of homogenously distributed species, only the most abundant species which cumulatively contributed to >90% of the biomass of each assemblage were included in our experimental simulation of extinction. Rare species were removed from all the treatments used to test species richness–ecosystem function relationships. However, control plots in which no species were removed were included in each manipulation as a reference. All biological material removed during manipulations was stored and later quantified along with the remaining biological material following measurements of ecosystem function to make estimates of species population dry Weight. In the case of salt marsh plants it was our intention to continue running the manipulation to monitor compensatory responses over time. Estimates of population dry weight were therefore estimated for each species in each plot by multiplying the % cover of each species by the average dry weight per unit % cover obtained from five of the control plots in which biomass was collected following the disturbance experiment conducted in 2008.

### Measuring Ecosystem Processes

Ecosystem processes were measured across the resulting gradients of species richness in each of the natural assemblages. Gross community productivity was measured as the rate of CO_2_ uptake in salt marsh plants. Gross community productivity was also measured in the macroalgae turf assemblage using O_2_ evolution in addition to the uptake of the key nutrients ammonium and nitrate. Clearance rates of mixed microalgae cultures (cell size range 1 to 20 um diameter) were measured for the sessile invertebrate assemblage.

Gross community productivity was measured in salt marsh plants using a LICOR LI840 CO_2_/H_2_O gas analyzer linked to a 30×30 cm clear plexiglass incubation chamber. CO_2_ uptake during photosynthesis and output through respiration were first measured during a light measurement (net community productivity). CO_2_ output from respiration was then measured independently during a dark measurement (community respiration). Gross community productivity was then estimated as net community productivity minus community respiration.

Gross community productivity and nutrient uptake of macroalgal assemblages were quantified using a 25×25 cm mesocosms on Menai Bridge Pier. Each mesocosm was filled with 5.2 l of fresh seawater from the Menai Strait prior to the estimation of both nutrient fluxes and gross community productivity. Hence initial concentrations of oxygen and nutrients were those of natural sea water at the time. 20 ml nutrient samples (filtered through a 0.45 um GF Whatman filter into acid rinsed bottles) were taken in duplicate 10, 50 and 90 minutes following the addition of algae with water being stirred prior to each measurement. On completion all samples were immediately transported back to the laboratory and stored at −20°C for later analysis. Nutrient samples were later analysed in the laboratory to quantify fluxes of Ammonium (NH_4_^+^) and Nitrate (NO_3_^−^). Nitrate concentrations were determined using an A5X-500 Series XYZ Auto Sampler (Zellweger analytics). Ammonium concentrations were determined fluorometrically using an F-2000 Fluorescence Spectrophotometer (Hitachi). The rate of nutrient flux was determined as the slope of the relationship between concentration and time for each of the nutrients analysed and expressed in µmol hr^−1^. Gross community productivity was estimated as O_2_ flux in mg O_2_ min^−1^ using two calibrated HACH LD40 probes following the method outlined by Noel *et al*., [44]. O_2_ utilization during community respiration was first quantified by immediately covering mesocosms in blacking out fabric removing 100% of available light. O_2_ concentration was measured ∼30 and 50 minutes later. Following dark measurements the blacking out fabric was removed and net community productivity (NCP) estimated by sampling O_2_ concentration a further 70 and 100 minutes later. Gross community productivity was then estimated as net community productivity–community respiration. Average light levels during the light measurement of net community productivity were 611.5±63.03 µmol m^−2^ s^−2^ PPFD.

Clearance rates of microalgae in suspension were estimated for sessile invertebrates under laboratory conditions. Tiles were placed in circular 2l tanks containing 0.1 µm filtered seawater at constant temperature and fasted for 24 hrs. Mixed microalgae cultures comprising 5 different species (*Nannochloropsis* sp., *Isochrysis* sp., *Pavlova* sp., *Tetraselmis* sp., and *Thalassiosira weissflogii*, Varicon Aqua) ranging in size from 1 to 20 µm cell diameter were added to give initial cell concentrations of 1.6×10^−5^±0.4×10^−5^ SE cells ml^−1^ g^−1^ community dry tissue weight. The range of microalgae cell sizes provided a resource that the communities were expected to partition as different groups of sessile invertebrates are well demonstrated in having contrasting optimum cell size ranges which they feed on [45], [46], [47], [48]. The 0 species treatment consisted of a bare PVC tile placed within the clearance rate tanks. 30 ml seawater samples were taken at regular time intervals of 5, 10, 15, 20, 30 and preserved in 2% Lugol's iodine pending analysis. Tanks were stirred continuously at 60 revolutions per minute throughout the clearance rate assays using mechanical stirrers to prevent microalgae settlement. Total cell concentrations were later estimated using a Coulter Multisizer II. Community clearance rate was estimated using the equation of [49] [*CR_tot_* = *V*(*a*-*b*)] where *a* is the rate of decline in each test suspension and *b* is the average rate of decline in log transformed particle concentration recorded from the 3 replicate 0 species treatments. *b* is subtracted from all values of *a* to account for any gravitational settlement of cells. *V* is the volume of the test suspension.

### Statistical Analysis

The rate of decline in each measured ecosystem process was estimated by fitting a variety of linear (linear and exponential) and non-linear (Michelis-Menten, Asymptotic and Log-logistic) models to each relationship and selecting the optimum model fit. In order to select the optimum model fit, first all models were tested for significance by comparison to a null (intercept only) model using Analysis of Variance. Second, of those models which were significantly different from the null, that which displayed the lowest value of Akike's Information Criterion (AIC) was selected as the optimum model fit. AIC provides a score which relates to how parsimoniously the model fits the data in a trade off between goodness of fit, and the number of parameters in the model. The lower the AIC value, the more parsimonious the fit. Non-linear models were fitted using the CRAN package nlrwr in the statistics platform R [50].

Functional contributions of species were estimated as the difference in the fitted values of the selected model between the two treatments where that species was lost. These values were then standardised to percentages of the summed value of all functional contributions (i.e. maximum ecosystem functioning) in each community to provide values of % functional contribution. % Contribution to community biomass was estimated from the average population biomass (dry weight) of species across all experimental units used to derive the species richness-ecosystem functioning relationship. The relationship between % functional contribution and % contribution to community biomass was then analysed using OLS regression. Because dominant species had a large leverage effect on the observed relationship, the analysis was repeated on 4^th^ root transformed data. To establish whether the relationship between % functional contribution and % biomass contribution was significantly different between ecosystems or ecosystem processes, a separate Analysis of Covariance was performed in each case. Residual normality was checked using Anderson-Darling tests and homogeneity of variance checked using Levene's test.

### Modelling Density Compensation

In the model of the best case scenario, it was assumed that at each stage of community disassembly, the extant species with the highest *per capita* (unit biomass) contribution to an ecosystem process fully compensated for any decrease in community biomass associated with species loss so that community biomass remained constant throughout the extinction sequence. To estimate the *per capita* functional contribution of each species, the functional contribution of each species was divided by the average population dry weight of that species recorded across those experimental plots in which community disassembly was initially simulated. The functional contribution of a species had previously been estimated from the fitted relationships between species richness and ecosystem functioning (Figure 1) as the decrease in the fitted model values of an ecosystem process as that species is lost from the ecosystem. Compensation was modelled by first sequentially removing species population biomass values from the community in sequence of the derived extinction orders. The biomass of the extant species at each stage of community disassembly with the highest *per capita* contribution to an ecosystem process was then artificially increased so that overall community biomass remained constant across all levels of species richness. The resulting population biomass values were then multiplied by the calculated *per capita* functional contribution of each species and totalled to provide an estimate of ecosystem functioning at each stage of community disassembly where the species with the highest *per capita* contribution always fully compensates for biomass loss associated with extinction, a best case scenario. Ecosystem functioning was expressed as a percentage of the maximum fitted value (i.e. the value at the highest level of species richness) derived from the original relationship between species richness and ecosystem function in the absence of density compensation (the worst case scenario, Figure 1). The resulting relationship was compared with the original species richness-ecosystem functioning relationship by estimating the number of species required to maintain each level of ecosystem functioning from 0 to 100%.

## Supporting Information

Figure S1 **The extinction resistance of species in three contrasting ecosystems undergoing disturbance.**
(PDF)
