# Supplementary material for: Dominance, Biomass and Extinction Resistance Determine the Consequences of Biodiversity Loss for Multiple Coastal Ecosystem Processes
Source: PLoS One. 2011 Dec 7;6(12):e28362. doi: 10.1371/journal.pone.0028362 (PMC3233569; doi:10.1371/journal.pone.0028362)
Supplement: Figure S1 — The extinction resistance of species in three contrasting ecosystems undergoing disturbance. (PDF) [file pone.0028362.s001.pdf]

**Figure S1. The extinction resistance of species in three contrasting ecosystems undergoing disturbance.**

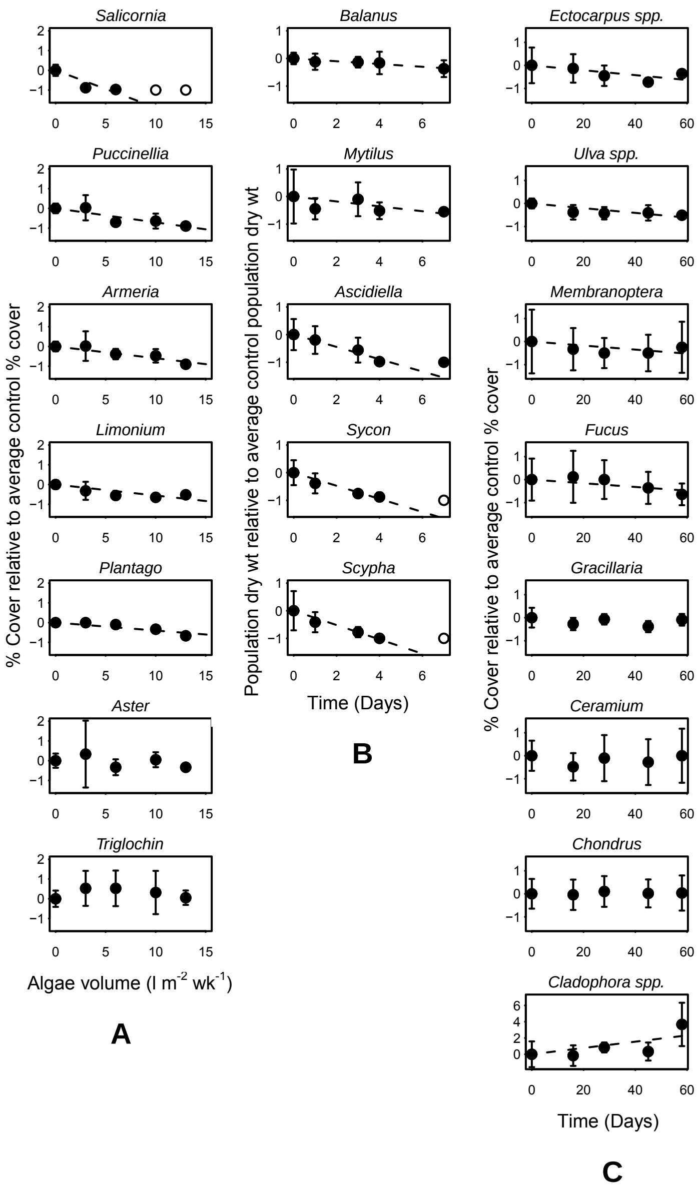


**Figure S1 The extinction resistance of species in three contrasting ecosystems undergoing disturbance.** A. The response of salt marsh plants to deposition of fucoid algae mats on the salt marsh surface. B. The response sessile suspension feeding invertebrates to the onset of hypoxic conditions. The response was measured as the rate of decrease in population biomass over time. C. The response of macroalgae species to increased wave expose mimicked by repeatedly pressure hosing the community. The response was measured as change in % cover through time. All responses on the y axis are expressed as relative to the average abundance of each species in the control (0 x axis) treatment. Values of 0 represent no change in the abundance of a species relative to the control, values of -1 represent complete removal of a species from the ecosystem, positive values represent an increase in the abundance of a species relative to the control. Dashed lines represent significant relationships bewtween distanbance intensity/duration and the abundance of a species. Species are ranked in order of their extinction resitsnace according to the slope of the relationship with the least extinction resistant species displaying more negative slope coefficients. Open points were omitted from the analysis to avoid underestimating the regression coefficients.
